# Rheology–Microstructure Relationships in Melt-Processed Polylactide/Poly(vinylidene Fluoride) Blends

**DOI:** 10.3390/ma11122450

**Published:** 2018-12-03

**Authors:** Reza Salehiyan, Suprakas Sinha Ray, Florian J. Stadler, Vincent Ojijo

**Affiliations:** 1 DST-CSIR National Centre for Nanostructured Materials, Council for Scientific and Industrial Research, Pretoria 0001, South Africa; VOjijo@csir.co.za; 2 Department of Applied Chemistry, University of Johannesburg, Doorfontein, Johannesburg 2028, South Africa; 3 College of Materials Science and Engineering, Shenzhen Key Laboratory of Polymer Science and Technology, Guandong Research Center for Interfacial Engineering of Functional Materials, Nanshan District Key Lab for Biopolymers and Safety Evaluation, Shenzhen University, Shenzhen 518055, China; fjstadler@szu.edu.cn

**Keywords:** PLA/PVDF blend, rheology, processing-driven morphology

## Abstract

In this study, small amplitude oscillatory shear tests are applied to investigate the rheological responses of polylactide/poly(vinylidene fluoride) (PLA/PVDF) blends and to correlate their viscoelastic properties with the morphological evolutions during processing. Although the analysis of the elastic moduli reveals some changes as a function of blend composition and processing time, the weighted relaxation spectra are shown to be more useful in detecting changes. The analysis demonstrates that when PVDF, i.e., the more viscous phase, is the matrix, the blend relaxes cooperatively and only a single relaxation peak is observed. By contrast, blends with highly concentrated morphologies do not fully relax, showing instead an upward increasing trend at longer times. This outcome is attributed to the broad distribution of highly concentrated droplets with a high probability of droplet–droplet contacts. Dynamic mechanical analysis (DMA) reveals that crystalline segmental motions attributed to the α-relaxation of PVDF at around 100 °C are restricted by the highly concentrated morphology of the 50/50 PLA/PVDF blend processed for 10 min. Relaxation analyses of the blends via dynamic oscillatory shear tests and DMA are shown to be powerful tools for investigating small microstructural changes in immiscible polymer blends.

## 1. Introduction

The blending of two or more polymers is an economically efficient strategy for the development of materials with interesting characteristics. However, most chemically different polymers are immiscible, and their blending leads to phase-separated morphologies with weak interfacial adhesion and thus poor mechanical performances. [1]. Since the final properties of the blends are directly dependent on the morphologies, many attempts have been made to tune the morphologies towards the desired application. The studies reported to date have shown that various morphologies can be developed by simply altering the dynamic and kinetic parameters, such as the blend ratio [2,3,4], the viscosity ratio [5], compatibilization [6,7,8], the mixing time [9,10], and the mixer type [11,12]. In recent years, the interest in bio-based polymer blends as alternatives to fuel-based ones has been growing as a result of the environmental issues that are associated with fuel-based blends [13]. Among the bio-based polymers available, polylactide (PLA) has attracted significant attention owing to its green characteristics (bio-degradable and biomedical) and reasonable modulus. However, its brittleness and slow crystallization rate have limited its application and processability [14,15]. Thus, PLA has been blended with other polymers with higher flexibility and lower rigidity to overcome such challenges [14,16,17,18,19,20]. In the previous study [21], an important benefit was found by blending PLA with poly(vinylidene fluoride) (PVDF); specifically, the addition of PVDF promoted the crystallization of PLA; for example, the mixing of 30 wt % of PLA with 70 wt % of PVDF for 10 min resulted in the formation of β-phase crystals. This result indicates the importance of morphological changes on the final properties of the blends.

The rheological properties of this particular blend have been studied previously [22,23]. Wang et al. [23] studied the rheological properties of the compatibilized and uncomaptibilized poly(l-lactic acid) (PLLA)/PVDF blends at different ratios via small amplitude oscillatory shear (SAOS) tests. As expected, the viscoelastic properties of the blends increased when the loading of the high-molecular-weight PVDF was increased. In their study, the authors reported a co-continuous morphology at a (65/35) PVDF/PLLA blend ratio, where the longest relaxation time ascribed to the relaxation of the droplets did not appear as a peak. In another study, Xie et al. [22] investigated the SAOS behavior of PVDF-rich PLA/PVDF blends with a maximum of 20 wt % of PLA. This work showed that increasing the PLA content from 5 to 15 wt % increases the elastic modulus as a result of the increased interfacial interactions between PVDF and PLA.

In the current work, for the first time, the rheological properties of PLA/PVDF blends were investigated exclusively with respect to the morphological changes occurring as a function of processing time and ratios in both PLA-rich and PVDF-rich compositions. The findings from the previous study [21] revealed that morphologies and properties are not determined solely by the composition ratios but also by the processing time, which can alter the properties particularly when the blends are not compatibilized. Therefore, rheological tools were utilized to identify the extent of the morphological changes induced by the processing conditions. The dependency of the droplet coalescence–breakup cycle on the viscosity ratio of the blends has been discussed. Continuous relaxation spectra of the blends were employed to analyze the morphological evolutions of the blends and droplet deformations and understand the influence of microstructural changes on the molecular dynamics of the blends. The results of this study will make a significant contribution to the literature because it demonstrates that experimental conditions, such as processing time and composition ratio, can be used to alter the properties of immiscible polymer blends.

## 2. Materials and Methods

### 2.1. Materials

Both the PLA and PVDF polymers used in this study were of commercial grade. The 7032D PLA (melt-flow index (MFI) = 13.81 g/10 min at 190 °C and 2.16 kg load), with a specific gravity of 1.24, was purchased from NatureWorks, LLC (Minnetonka, MN, USA). PVDF (MFI = 2.2 g/10 min at 300 °C and 3.8 kg load), with a density of 1.74 g/cm^3^ at 20 °C, was obtained from SynQuest Labs., Inc. (Alachua, FL, USA). The MFI values per conditions shown indicate the very high viscosity of PVDF when compared to PLA. Both polymers have similar melting point ranges (155–170 °C) according to the suppliers and findings from a previous study [21].

### 2.2. Processing

Pellets of PLA and PVDF were kept in a vacuum oven at a temperature of 70 °C for about 12 h prior to mixing in order to remove excess moisture. The dried hand-mixed ratios (70/30; 50/50; and 30/70) of PLA/PVDF mixtures were melt-blended in HAAKE PolyLab OS Rheomix (Thermo Fisher Scientific, Dreieich, Hessen, Germany) at a rotor speed of 60 rpm and a temperature of 190 °C for 5, 7.5, and 10 min. Subsequently, the blends were compression-molded using a Carver Laboratory hot press (Wabash, IN, USA) at 190 °C and cooled to room temperature to prepare discs with a diameter of 25 mm for rheological characterization.

### 2.3. Rheometry

Samples were annealed at 60 °C for 12 h before the rheological characterization in order to avoid the effects of moisture trapped in the bulk of the samples on the results. A Physica MCR501 rheometer (Anton Paar, Graz, Austria) with 25-mm-diameter parallel plates was used to perform rheological analyses under a nitrogen environment. SAOS tests were carried out for frequencies ranging from low (0.01 rad/s) to high (100 rad/s) at a fixed strain of 0.5% (linear region) and a temperature of 200 °C.

### 2.4. Determination of Continuous Relaxation Spectra

The continuous relaxation spectra were determined according to the method reported by Stadler and Bailly [24], which, unlike all other methods, does not calculate the spectrum directly but instead optimizes *n* spectra descriptors—knots of a Hermite spline—which are subsequently used to determine the spectrum in the form of a Hermite spline, and used to reconstruct the input data (*G*′(*ω*) and *G*″(*ω*)) from the spectrum as follows:

(1) $$G^{\prime }\left(\omega \right)=G_{\infty }+\overset{n}{\underset{i=1}{\sum }}g_{i}\left(\tau_{i}\right)\cdot \left(\frac{\omega^{2}\cdot \tau_{i}^{2}}{1+\omega^{2}\cdot \tau_{i}^{2}}\right)=G_{\infty }+\int_{0}^{\infty }H\left(\tau \right)\cdot \left(\frac{\omega^{2}\cdot \tau^{2}}{1+\omega^{2}\cdot \tau^{2}}\right)d\ln\tau$$

(2) $$G^{\prime \prime }\left(\omega \right)=\overset{n}{\underset{i=1}{\sum }}g_{i}\left(\tau_{i}\right)\cdot \left(\frac{\omega \cdot \tau_{i}}{1+\omega^{2}\cdot \tau_{i}^{2}}\right)=\int_{0}^{\infty }H\left(\tau \right)\cdot \left(\frac{\omega \cdot \tau }{1+\omega^{2}\cdot \tau^{2}}\right)d\ln\tau$$

The spectrum descriptors and, consequently, the spectrum were optimized using a pursuit algorithm to minimize the least mean square error *D*^2^* according to the formula reported by Baumgärtel and Winter [25], with the addition of the slope penalty *s*:

(3) $$D^{2*}=\sqrt{\frac{1}{n}\overset{n}{\underset{i=1}{\sum }}\left[{\left(\frac{G_{i}^{\prime }-G_{i\;\mathrm{fit}}^{\prime }}{G_{i}^{\prime }}\right)}^{2}+{\left(\frac{G_{i}^{\prime \prime }-G_{i\;\mathrm{fit}}^{\prime \prime }}{G_{i}^{\prime \prime }}\right)}^{2}\right]\;}+s$$

which forbids excessively large slopes, and is defined as:

(4) $$s=\int_{\tau_{i-1}}^{\tau_{i}}zd\tau ,\;\mathrm{with}\;Z(\tau )\;=\;10,\;\mathrm{if}\;\frac{d\log H}{d\log\tau }\left(\tau \right)>m,\;\mathrm{and}\;Z(\tau )\;=\;0,\;\mathrm{if}\;\frac{d\log H}{d\log\tau }\left(\tau \right)\leq m$$

with *m* being set to 2, i.e., a value that reliably avoids excessive slopes and at the same time will not influence the spectrum. This slope penalty was shown to avoid unphysical solutions arising from the pursuit algorithm getting stuck in local minima of *D*^2^*.

In order to properly model the data, we first determined the range of relaxation times that has a significant influence on the input data by varying the relaxation time range in which the spectrum is calculated, which was subsequently analyzed using the integrals of the relevance factors as defined previously [26]. After pre-tests, we found that the spectrum can be determined successfully in a range that is one order of magnitude higher than the input data and the input data can be very successfully described with the determined spectrum; hence, an inverse input data interval plus one decade in each direction was used for all calculations presented here.

### 2.5. Morphological Analysis

The morphologies of the blends were studied using a Carl Zeiss Auriga (Oberkochen, Germany) scanning electron microscope (SEM) on cryo-fractured surfaces of the molded samples. The number average ($R_{n}$) and volume average ($R_{v}$) droplet sizes and size distributions were calculated according to Equations (5)–(7), respectively. The radii of over 100 droplets were calculated for each sample from three different images using the image analysis software ImageJ (1.46r, National Institute of Health, Bethesda, MD, USA).

(5) $$R_{n}=\frac{\sum n_{i}R_{i}}{\sum n_{i}}$$

(6) $$R_{v}=\frac{\sum n_{i}R_{i}^{4}}{\sum n_{i}R_{i}^{3}}$$

(7) $$D=\frac{R_{v}}{R_{n}}$$

In which $n_{i}$ is the number of droplets with radius $R_{i}$.

### 2.6. Dynamic Mechanical Analysis

A PerkinElmer dynamic mechanical analyzer (DMA, Waltham, MA, USA) 8000 was used in a single bending mode to carry out the dynamic mechanical analysis of the 10-min-processed blends. Thin films of the (29–30 mg) samples were placed inside the solid steel material pockets. The modulus and tan δ of the blends were measured from −60 to 140 °C at a rate of 2 °C/min, a frequency of 1 Hz, and a strain amplitude of 0.05%.

## 3. Results

### 3.1. Small Amplitude Oscillatory Shear Tests

**Figure 1:**
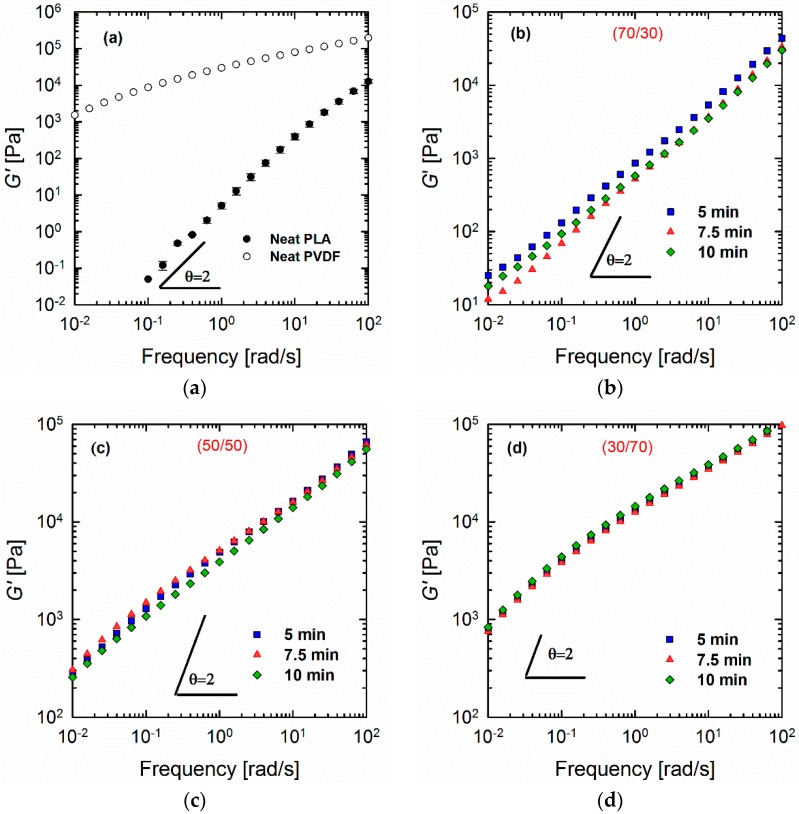

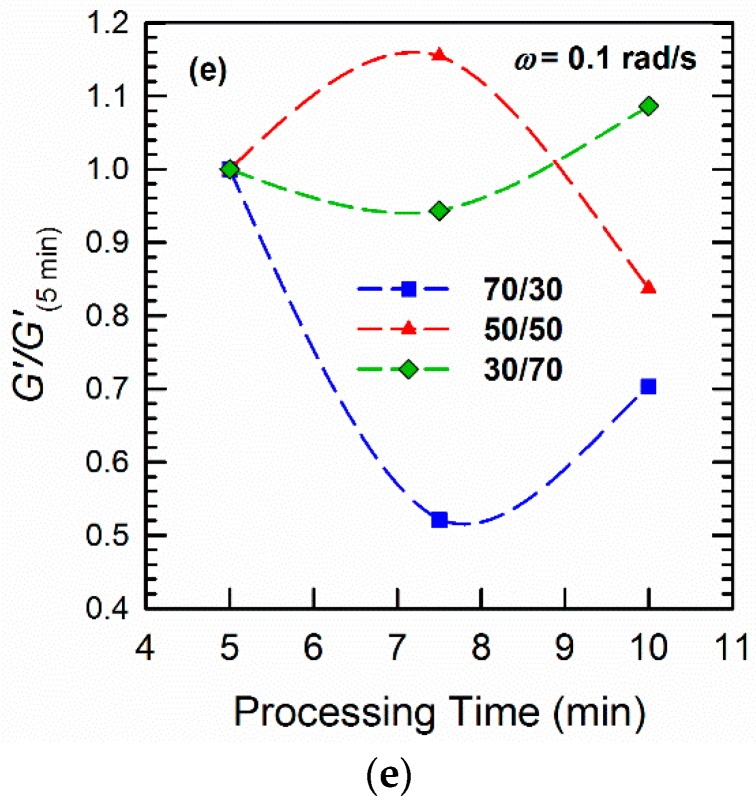
Storage moduli *G*′(*ω*) of (**a**) neat polymers and (**b**) 70/30, (**c**) 50/50, and (**d**) 30/70 PLA/PVDF blends processed for different times as a function of frequency from 0.01 to 100 rad/s at strain amplitude of 0.5% and a temperature of 200 °C under a nitrogen atmosphere; (**e**) storage moduli of the blends normalized relative to the storage moduli of the blends processed for 5 min at a fixed frequency of 0.1 rad/s.

**Figure 2:**
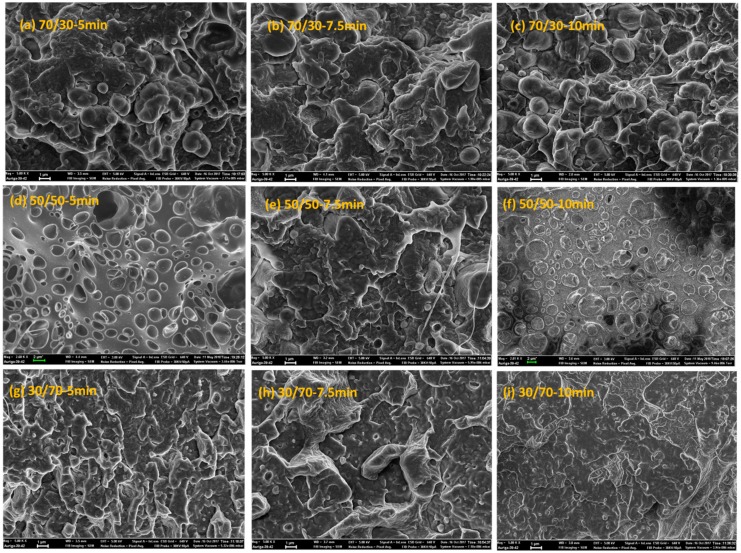
SEM images of (**a**–**c**) 70/30, (**d**–**f**) 50/50, and (**g**–**i**) 30/70 PLA/PVDF blends processed for (**a**,**d**,**g**) 5 min, (**b**,**e**,**h**) 7.5 min, and (**c**,**f**,**i**) 10 min. Scale bars are always equal to 1 µm, with the exception of (**d,f**), where the scale bars indicate 2 µm. This figure is reprinted with permission from [21].

**Figure 3:**
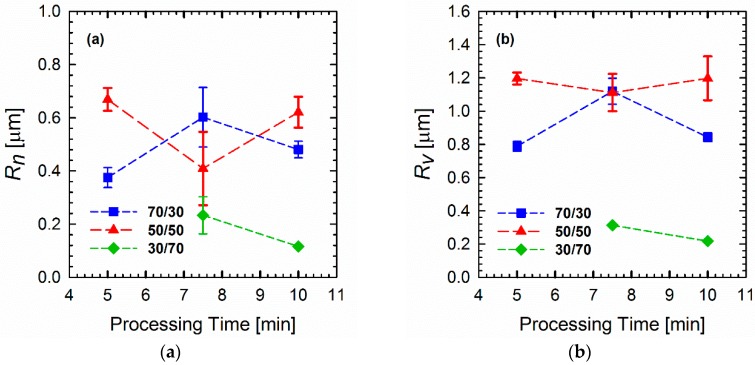
(**a**) Number average (*R_n_*) and (**b**) volume average (*R_v_*) radii sizes of droplets in blends of various compositions calculated based on Equations (5) and (6) from SEM images. The 30/70 PLA/PVDF blend processed for 5 min did not show a distinct droplet morphology, and, hence, the calculations for this composition were done after processing for 7.5 and 10 min only.

Figure 1 shows the elastic moduli *G*′(*ω*) of the neat polymers and polymer blends of different compositions and processing times. It is apparent that neat PLA follows the terminal behavior with a power law slope of 2 (for simplicity, the slopes are always mentioned as apparent slopes in the scaling, in which they are plotted, i.e., Figure 1a shows a power law slope of 2 (dlog*G*′/dlog*ω* = 2), which is referred to as slope of 2). The loss moduli *G*″(*ω*) and complex viscosities |η*(*ω*)| of the blends are detailed in the Supplementary (Figures S1 and S2, respectively). By contrast, PVDF deviates remarkably from such classical behavior, which is indicative of a very large elasticity and a slow relaxation process. These observations are most likely related to the chain mobilities of the polymers. As will be discussed later (see the Section on DMA), PVDF has a glass transition temperature (β-relaxation) of around −30 °C. The weighted relaxation spectra of the polymers and blends also demonstrated the long relaxation mechanism of PVDF and PVDF-rich blends (see the Section on relaxation spectra). As shown (Figure 1), the responses of all of the blends were somewhere between those of the two neat polymers, obeying the simple rule of mixtures. It is interesting to observe the differences in morphological changes during the processing in a blend at a fixed composition (Figure 2). Such morphology development can be explained by the mechanism of droplet deformation during the processing. It is known that the final morphology of an immiscible polymer blend is governed by the balance between droplet breakage and coalescence [27,28]. That is, both droplet collision and deformation occur during the mixing process concurrently [29]. Figure 1 shows that this phenomenon also depends on the composition ratio. In the case of the 70/30 PLA/PVDF blends (Figure 1b), the storage modulus *G*′(*ω*) is higher when the blend is processed for 5 min. Processing of the blend for a further 2.5 min (overall 7.5 min) decreased the modulus to its minimum value. This result is caused by the fact that highly viscous PVDF droplets are less likely to break further at this point, and, hence, droplets collide to form bigger droplets. The formation of larger droplets is accompanied by a decrease in the modulus. Eventually, the processing for 10 min allowed the coalesced droplets to break up again, which manifested in a slight increase in the modulus. On the other hand, in the case of 50/50 PLA/PVDF blends, the optimized processing time was established as 7.5 min. Since the two phases are present in equal amounts, one can expect larger domains, and, hence, different collision–breakup frequencies. Finally, increasing the viscosity of the matrix by changing the blend ratio to 30/70 PLA/PVDF has reduced the breakup time; consequently, the subsequent collisions also occurred faster. For this reason, the differences observed for the blend with a 50/50 composition as a function of processing time were less obvious. In order to develop a better understanding of these differences, the moduli of the blends normalized relative to the moduli of the 5-min-processed blends were plotted as a function of processing time for a fixed frequency of 0.1 rad/s (Figure 1e). Since the differences are more significant at longer times (lower frequency regions), the moduli at 0.1 rad/s were selected to observe the changes as a function of processing time. From Figure 1e, it can be observed that the changes become more significant as the PLA concentration increases. That is, when hard spheres of PVDF exist as the minor dispersed phase within the PLA matrix, the probability of coalescence is higher; or, in other words, the coalescence–breakup cycle is quite slow. By contrast, when PLA is the minor phase within the PVDF matrix, the break-up into smaller droplets occurs more readily, and these can again coalesce into bigger droplets since the blend is not compatibilized. Therefore, a shorter coalescence–breakup cycle can be expected, and consequently, the changes observed are less significant. The results are in agreement with the SEM images of the blends, shown in Figure 2. It must be noted that all of the studied blend ratios exhibited a droplet-matrix morphology. The number average (*R_n_*) and volume average (*R_v_*) droplet radii sizes of the blends are shown in Figure 3a,b, respectively.

### 3.2. Van Gurp–Palmen Plots

**Figure 4:**
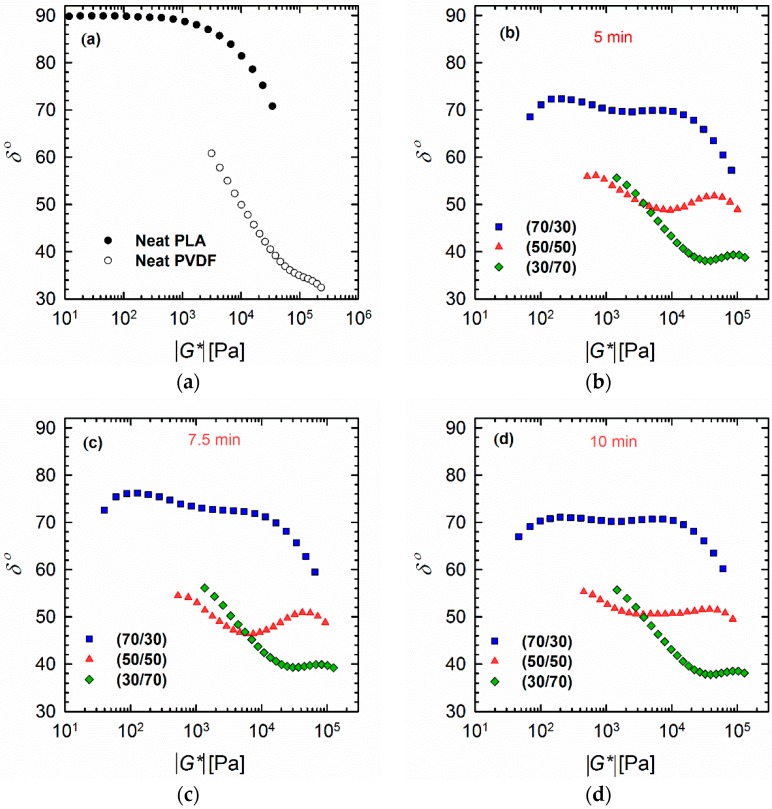
Van Gurp–Palmen plots of (**a**) neat polymers and (**b**–**d**) 70/30, 50/50, and 30/70 PLA/PVDF blends processed for (**b**) 5, (**c**) 7.5, and (**d**) 10 min at 200 °C.

The plots of phase angle *δ* as a function of absolute complex modulus |G*(*ω*)|, also known as van Gurp–Palmen plots (Figure 4), can also be considered as a complementary tool to investigate the morphology of the polymer blends [30,31,32]. From these plots, it is clear that PLA exhibits behavior that is typical for linear polymers, where the value of the phase angle *δ* reaches a plateau region (90°) at lower |G*(*ω*)| values. By contrast, PVDF shows an immediate decrease in *δ* values starting around 60°; this decrease continues until a *δ* of ~35°, at which point a small hump with higher |G*(*ω*)|values is observed (Figure 4a). This behavior could be caused by the much higher viscosity and entanglement of the PVDF, which mean that it did not fully relax. It has also been reported previously that a valley at low |G*(*ω*)|values indicates a droplet–matrix morphology, while a maximum at low |G*(*ω*)|regions is a sign of co-continuous morphology [30]. However, Lopez-Baron and Macosko [31] showed that this rule may not be applicable to all systems. The current vGP plots (Figure 4b–d) revealed shapes that are completely different from those present in previously reported studies; this discrepancy probably stems from the complex structure of PVDF. The blends (particularly the 70/30 and 50/50 PLA/PVDF blends) showed bimodal behavior, where two distinct peaks can be observed. This phenomenon can be related to the relaxation of each phase. In the case of the 70/30 PLA/PVDF blend, the peaks are quite distinct and correspond to the distribution of PVDF droplets in the PLA matrix. Further, the first maximum in the case of the 50/50 blend has decreased at 7.5 min, and faded completely after 10 min, which indicates the long relaxation of PVDF associated with very low frequencies. This result is in accordance with the relaxation spectra of the blends, and could be related to the highly close-packed morphology (i.e., the high concentration of droplets) of this 50/50 blend, as evidenced by the SEM images. In the case of the 30/70 PLA/PVDF blend, the *δ* values decreased monotonically on going from 55° to 38° (valley area), and subsequently exhibited a small hump related to the relaxation of the PLA phase. This hump again confirms the complexity of the morphologies of this particular blend and the dominance of PVDF relaxation in PVDF-rich blends.

### 3.3. Cole–Cole Plots

**Figure 5:**
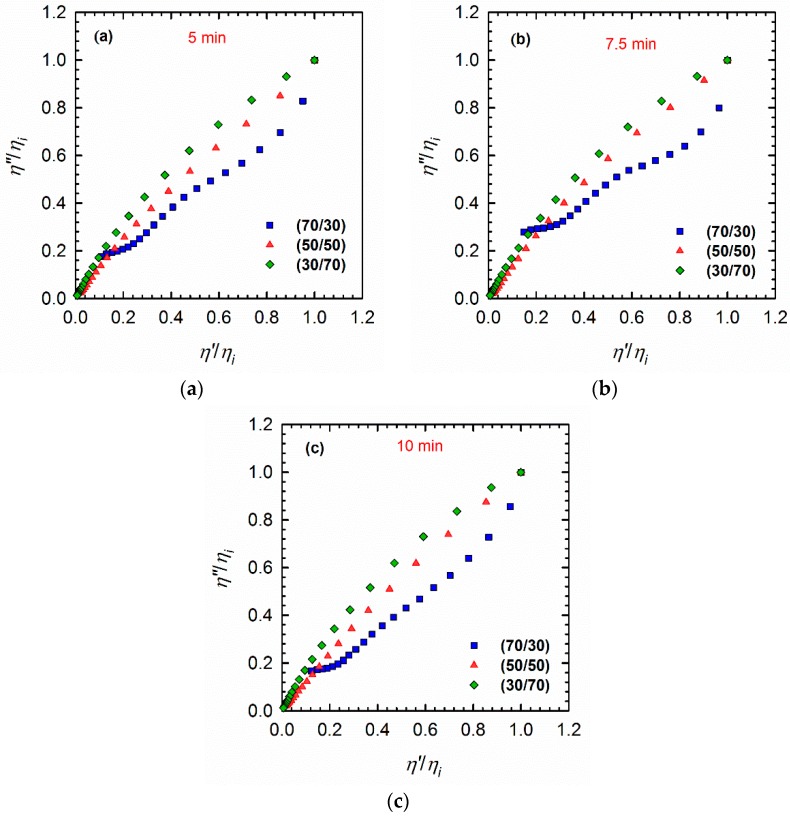
Cole–Cole plots of PLA/PVDF blends of different compositions processed for (**a**) 5 min, (**b**) 7.5 min, and (**c**) 10 min. The plots are normalized relative to the values of *η*′ and *η*″ of the respective initial viscosities *η_i_*.

**Figure 6:**
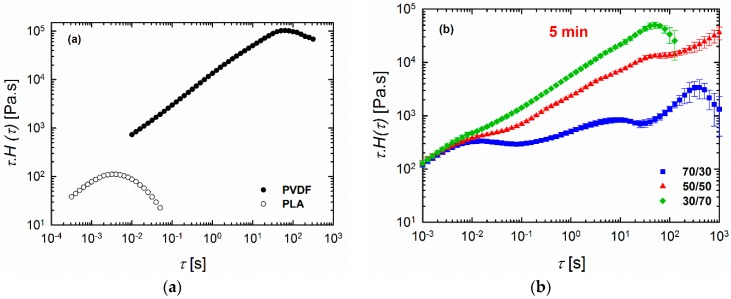

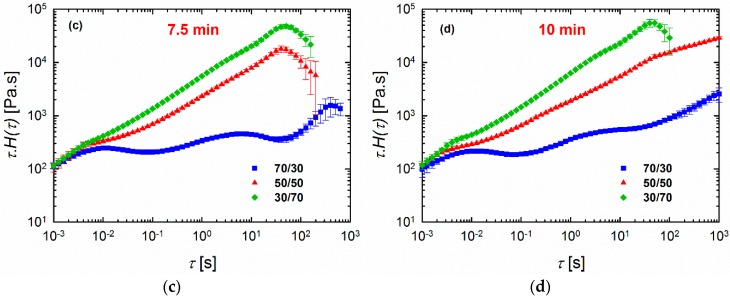
Weighted relaxation spectra of (**a**) neat polymers and (**b**–**d**) 70/30, 50/50, and 30/70 PLA/PVDF blends processed for (**b**) 5 min, (**c**) 7.5 min, and (**d**) 10 min at 200 °C.

A plot of *η*″ versus *η*′, known as the Cole–Cole plot, is another tool that can be used to investigate the structures of phase-separated blends [33]. Parameters *η*″ and *η*′ are imaginary and real contributions of the complex viscosity (η* = *η*′ − *i*·*η* ″). According to the literature [30,31,34], polymers with monomodal molecular weight distributions show a typical arc-shape curve, where a double-arc shape indicates the droplet–matrix morphology. The first arc on the left-hand side of the plots (at low *η*′ regions) is associated with relaxation of the polymers, whereas the arc on the right-hand side of the plots (at higher *η*′ values) represents the relaxation of the droplets. The appearance of a tail at high *η*′ values is known to be attributed to the interface, and indicates a co-continuous morphology. Taking this information into account, the blends under investigation in this study revealed different behaviors (Figure 5). Specifically, the 70/30 PLA/PVDF blend exhibited a slight hump that indicates the droplet–matrix morphology; by contrast, the 50/50 and 30/70 PLA/PVDF blends displayed a monotonic increase from low to high *η*′ values. This increase could be caused by the nonlinear molecular structure of the PVDF, which makes these particular compositions cooperative segmental relaxations, as confirmed by analyses of the relaxation spectra (Figure 6).

The Cole–Cole plots confirmed the results from the vGP plots, although the differences in the blends processed for different times (Figure 5) remained practically indistinguishable.

### 3.4. Relaxation Spectra

**Figure 7:**
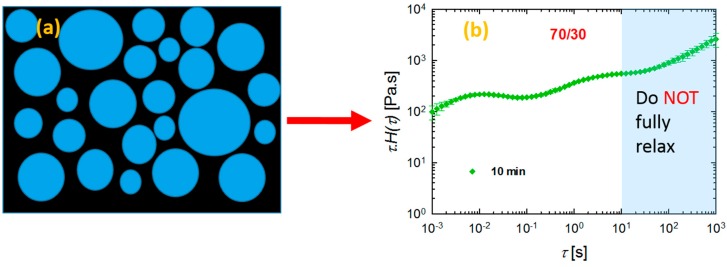
Schematic presentation of a relaxation spectra of a blend with a condensed morphology where the round-shaped domain phases are located in very close vicinity to one another as in the case of the 10 min processed (70/30) and 5 min and 10 min processed (50/50) PLA/PVDF blends.

The differences in rheological responses originate from the relaxation of the polymers under shear conditions, and, consequently, it is worthwhile to examine the relaxation spectra of the blends. It has been shown that analyses based on continuous relaxation spectra could be very useful in the characterization of microstructures of materials with multiphase structures, such as blends [24,35,36]. Therefore, the weighted relaxation spectra of the polymers and blends processed at different times are plotted in Figure 6. It is immediately apparent that PLA relaxes quite fast at around 10^−3^ < *τ* < 10^−2^ s, while PVDF relaxes quite slowly, with *τ* around 63 s (Figure 6a). It is clear that we have two polymers with completely different molecular structures and entanglement networks. Further, the blends also showed noticeably different behaviors. Among the blends examined, the 70/30 PLA/PVDF blend exhibited curve shapes that were quite different from the blends with 30/70 and 50/50 compositions. Three peaks were observed for this particular composition (70/30 PLA/PVDF). The peak at the very low relaxation time around *τ* = 0.01 s corresponds to the relaxation time that is characteristic for PLA chains; the peak beyond *τ* ≈ 100 s can be ascribed to the relaxation of PVDF. Moreover, the peak around *τ* ≈ 5 s is associated with the form (shape) relaxation of PVDF droplets. The droplets of the minor phase undergo deformation during the oscillatory shear test; hence, they reserve the imposed energy. Therefore, the energy required to retract the deformed droplets to their equilibrium spherical shape (shape relaxation) causes an enhancement in the storage modulus (elasticity). Taking this information into account, a more pronounced shape relaxation process, and thus higher elasticity, can be expected for the systems with droplets that are more susceptible to deformation under force. In other words, blends with large viscosity ratios *K* = *η_d_*/*η_m_* (where *η_d_* and *η_m_* are the viscosity of the dispersed phase and matrix, respectively) would have a lower amount of stored energy, and, thus, also shorter shape relaxation times as a result of the less deformed droplets. Therefore, as PVDF is a highly viscous phase, the droplets can hardly be deformed under the imposed dynamic oscillatory shear conditions, and, therefore, they exhibit relatively shorter relaxation times. A closer look at the SEM image of the 70/30 blend processed for 10 min (Figure 2c) reveals that the droplets are more in close-packed structures, which may promote cooperative relaxation processes and restrict interfacial relaxations. Hence, the second peak has become less pronounced in the 70/30 blend processed for 10 min. In contrast to the systems with a large viscosity ratio, the systems that have a viscosity ratio smaller than unity, as in the case of the 30/70 PLA/PVDF blends, exhibit droplets with a much lower viscosity dominated by PLA, which will deform readily under the shear. That is, a longer time and more energy are required to pull back the deformed droplet. Interestingly, when the concentration of the highly crystalline PVDF was increased (i.e., the 30/70 PLA/PVDF blend), the material exhibited only a single characteristic relaxation peak at around *τ* ≈ 50 s, similar to that of the neat PVDF. Previous studies have reported that such behavior in blends where polymers do not relax independently can be attributed to the cooperative relaxation mechanism where only one peak appears in the relaxation spectrum, and this peak is similar to that observed for the single polymer [37,38,39]. According to Saito et al. [39], such cooperative relaxations in a binary system can occur when dynamic contacts exist between the two polymers. It has been also reported that such cooperative relaxation processes at the interface can be constrained by crystalline lamellas [40]. Therefore, increasing the fraction of the more crystalline (high viscosity) phase, PVDF in this case, enhances the restrictions at the interface, and, therefore, also the relaxations. It is worth noting that, in the case of the 50/50 blend (particularly after processing for 5 min and 10 min), full relaxation was not observed. Consequently, no distinct peak could be observed, most likely as a result of the broad distribution of the domain phase and the very highly concentrated droplet morphologies (Figure 2c,d,f). In such a morphology, where a very populated droplet-shape minor phase exists (Figure 7), the interface cannot completely relax due to the constrained morphology. By contrast, in the case of the 50/50 blend processed for 7.5 min, a more visible peak could be observed, probably due to the slight morphological reformations (Figure 2e).

### 3.5. Interfacial Tension Analysis

**Figure 8:**
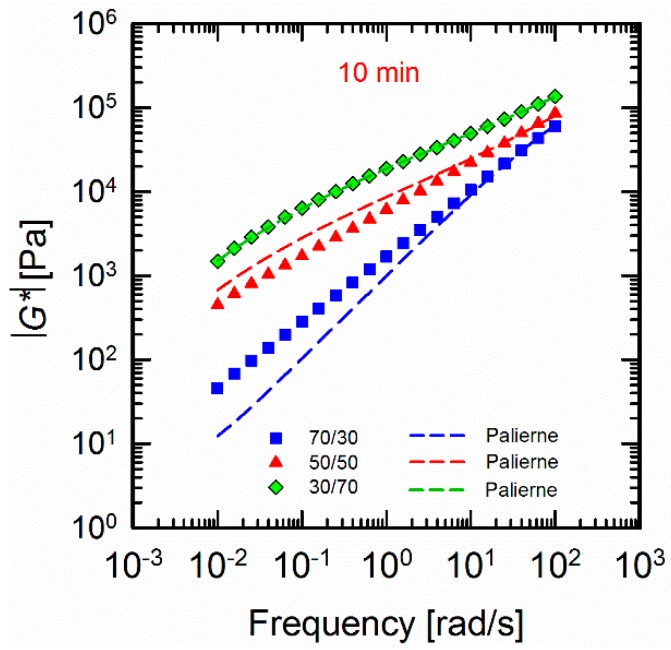
Palierne fittings of the 10 min processed (70/30), (50/50), and (30/70) PLA/PVDF blends measured at 200 °C. The dashed lines represent the fitted results.

The relaxation spectra of the blends revealed the importance of the role of the interface in defining the non-terminal behaviour of the blends at longer times. It has been reported that the increase in elasticity of the blends could be related to the development of interfacial areas [41,42,43]. The Palierne model [44] has extensively been utilized to study the interfacial properties of the uncompatibilized and compatibilized blends [20,45,46,47]. However, it must be noted that the Palierne model is restricted to low concentrated systems with spherical morphologies with no droplet–droplet interactions [47]. Therefore, discrepancies between theoretical predictions and experimental calculations are inevitable. In this study, we employed the Palierne model (Equation (8)) to fit the complex moduli $\left|G^{*}\left(\omega \right)\right|$ of the 10 min processed blends in parallel with the Gramespacher and Meissner model [43] (Equation (9)), respectively, to examine the interfacial tensions α. The Gramespacher and Meissner model was used based on the calculated relaxation spectra.

(8) $$G_{b}^{*}\left(\omega \right)=G_{m}^{*}\left(\omega \right)\frac{1+3\emptyset H^{*}\left(\omega \right)}{1-2\emptyset H^{*}\left(\omega \right)},\;H^{*}\left(\omega \right)=\frac{4\left(\frac{\alpha }{R_{v}}\right)\left[2G_{m}^{*}\left(\omega \right)+5G_{d}^{*}\left(\omega \right)\right]+\left[G_{d}^{*}\left(\omega \right)-G_{m}^{*}\left(\omega \right)\right]\left[16G_{m}^{*}\left(\omega \right)+19G_{d}^{*}\left(\omega \right)\right]}{40\left(\frac{\alpha }{R_{v}}\right)\left[G_{m}^{*}\left(\omega \right)+G_{d}^{*}\left(\omega \right)\right]+\left[2G_{d}^{*}\left(\omega \right)+3G_{m}^{*}\left(\omega \right)\right]\left[16G_{m}^{*}\left(\omega \right)+19G_{d}^{*}\left(\omega \right)\right]}$$

(9) $$\alpha =\left[\frac{R_{v}\eta_{m}}{\tau }\right]\left[\frac{\left(19K+16\right)\left(2K+3\right)}{40\left(K+1\right)}\right]\left[1+\emptyset \left(\frac{5\left(19K+16\right)}{4\left(K+1\right)\left(2K+3\right)}\right)\right]$$

where $G_{b}^{*}\left(\omega \right)$, $G_{m}^{*}\left(\omega \right),$ and $G_{d}^{*}\left(\omega \right)$ are the complex moduli of the blend, the matrix, and the dispersed phase at the angular frequency of ω, respectively. ∅, $R_{v}$, *K*, and α are the volume fraction of the dispersed phase, the volume-averaged droplet size of the dispersed phase, the viscosity ratio, and the interfacial tension, respectively. The fitted results are shown in Figure 8. The interfacial tensions calculated from both the fitting (Equation (8)) and the relaxation spectra (Equation (9)) are summarized in Table 1.

It can be observed that the Palierne model fails to predict the moduli of the (70/30) and (50/50) blends. It has also been reported that the Palierne model and its derivatives can be used for the systems with droplet distributions less than 2, where the system is not highly concentrated as in the case of (70/30) and (50/50). As we discused earlier, the 10 min mixed (70/30) and (50/50) blends showed highly populated morphologies that may encourage the droplet–droplet contacts, and, as a result, this can affect the calculations and cause uncertainty in the fitting results. It can also be seen that when PLA is the minor phase in (30/70) PLA/PVDF blends the model fits well. This reflects the narrow droplet size distributions in this particular composition. It can be inferred that the high viscos PVDF matrix immobilized the PLA droplets where a lower probability of the droplet–droplet interactions is provided. Instead, the low viscosity ratio has provoked the droplet breakup. As a result, morphologies with smaller droplets and larger surface areas are obtained. The large values of interfacial tensions obtained along with the ill-fitted results indicated that such models cannot be used to predict the interfacial tensions of the highly populated droplet morphologies. The estimations, however controversial, confirmed the lower interfacial tensions for the composition where the higher viscos phase PVDF is the matrix and the low viscos phase PLA, the dispersed phase.

Although the predicted values are rough estimations and, as explained above, they are affected by simplifications and droplet size measurements, they can be used as complementary results confirming the effect of the composition ratio and the viscosity ratio on the viscoelastic response and morphological evolutions of the blends. It must be also taken into account that the blends exhibited a somewhat cooperative relaxation process, which could influence the final calculated values.

### 3.6. Dynamic Mechanical Analysis

**Figure 9:**
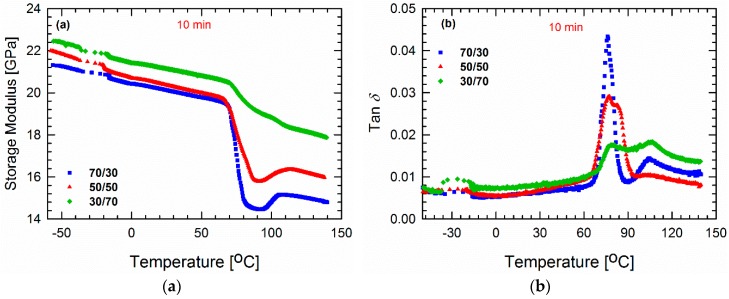
(**a**) Storage moduli *E*′ and (**b**) tan *δ* values of blends processed for 10 min as a function of temperature (−60 to 140 °C) and measured at a frequency of 1 Hz.

Thus far, we have focused on probing the importance of chain mobility and dynamics. At this point, it is of interest to observe the molecular dynamics of the polymers at different temperatures, and one promising method of doing so utilizes the DMA tests. The storage modulus *E*′ and the loss factor (tan *δ* = *E*″/*E*′ where *E*″ and *E*′ are the loss and storage moduli, respectively) values of the blends processed for 10 min as a function of temperature are plotted from −60 to 140 °C in Figure 9. According to literature reports, PVDF has a glass transition temperature Tg of around −30 °C [48,49,50]. Studies have shown that tan *δ* peak around −30 and 100 °C are associated with amorphous (β-relaxation) and crystalline (α-relaxation) segmental motions, respectively [51,52,53,54,55]. In Figure 9a, a rapid decrease in storage modulus around (70–80 °C) is attributed to the glass transition temperature of the blends, which further rises again around 90–110°C. This increase in storage modulus at a high temperature corresponds to the cold crystallization of PLA where, at a lower PLA concentration (30/70), it gradually fades away. A further decrease in modulus also indicates the onset of melting and softening of the blends [56,57]. Figure 9b reveals that the β-relaxation peak is more significant in the case of the 30/70 PLA/PVDF blend. Such β-molecular rearrangements are known to govern the crystal growth and nucleation process in a system [58,59]. By contrast, the peak around 76 °C corresponds to the Tg of PLA. It should be noted that the differences between the reported and measured values of Tg could be caused by the differences in heating rates, as well as the fact that during DMA tests, a force is imposed on the samples. As expected, it can be seen that the height of the peak associated with the Tg of PLA increases gradually as the PLA content increases. Interestingly, the α-relaxation peaks can only be seen in the case of the 70/30 and 30/70 PLA/PVDF blends, and no such peak is observed for the 50/50 blend. Instead, this blend exhibits a broader peak at the Tg of PLA. This result can be explained by the weighted relaxation spectra, where the 50/50 blend did not display a distinct peak at high relaxation times. As explained in the previous section, since this particular composition has the populated droplet-shape domain phase, the interface is constrained during the relaxation process. Therefore, the crystalline segments of PVDF cannot relax completely.

## 4. Conclusions

In this study, linear dynamic oscillatory tests were exploited to unravel the morphologies of PLA/PVDF blends at three different ratios (30/70, 50/50 and 70/30) and their dependency on the processing time. The rheological analyses based on SAOS tests revealed, as expected, elasticity enhancements in systems with increased PVDF content owing to the significantly higher viscosity of neat PVDF when compared to that of neat PLA. The analysis also revealed that, since the blends were not compatibilized, they underwent changes during the processing. The SEM images of the blends mirrored the rheological responses. In blends with the higher viscosity phase PVDF as the minor phase, the large viscosity ratio prevented the break-up process, and PVDF droplets instead coalesced during the processing, causing a slight drop in elasticity. In order to better understand the behaviors of the blends under shear tests, continuous relaxation spectra were calculated. The weighted relaxation spectra were quite useful in unraveling the microstructures of the blends. PVDF was found to have a large relaxation time, most likely arising from the presence of entangled networks. Therefore, in PVDF-enriched PLA/PVDF blends (i.e., 30/70 ratio), the spectra were affected by the very slow relaxation of PVDF, and exhibited a cooperative relaxation mechanism; consequently, only a single relaxation peak could be observed. In PLA-enriched PLA/PVDF blends (i.e., 70/30 ratio), the relaxation peak associated with the relaxation of PVDF was broad and found between 5 and 10 s. This result was attributed to the broad droplet distribution of the blend, since the high viscosity PVDF droplets are difficult to break into smaller ones. The analysis also showed that highly concentrated structures, e.g., those in the 50/50 blend, exhibited relaxation spectra that increased progressively, indicating a lack of full relaxation. This feature also manifested in the DMA results, where α-relaxation associated with the crystalline motions around 100 °C was reduced in the case of the 50/50 blend processed for 10 min. Generally, analyses based on relaxation mechanisms of the blends could give better insight into their microstructures. Therefore, we can conclude that, for immiscible polymer blends, an understanding of the correlation between a processing-driven morphology and rheological properties is crucial to gain knowledge on processibility and product development.

## Figures and Tables

**Table 1:** The interfacial tension predicted by the Palierne model and the Gramespacher and Meissner model for the 10 min processed blends.

Blend	* $\alpha \;\left[\mathrm{mN}/m\right]{\;}^{a}$	* $\alpha \;\left[\mathrm{mN}/m\right]{\;}^{b}$	τ (s)	$R_{v}\;\left(µm\right)$	$R_{n}\left(µm\right)$	D
(70/30)	14.44	86.27	5	0.84 ± 0.03	0.48 ± 0.04	1.76 ± 0.17
(50/50)	25.57	72.80	50	1.19 ± 0.18	0.62 ± 0.08	1.93 ± 0.21
(30/70)	5.12	9.86	50	0.21 ± 0.00	0.11 ± 0.00	1.86 ± 0.12

^a^ calculated from fitting the Palierne model; ^b^ calculated from weighted relaxation spectra (the Gramespacher and Meissner model); * for calculation of the viscosity ratio *K*, the Carreau–Yasuda model was used to fit the viscosity of the neat polymers since PVDF did not show the plateau behaviour (Figure S3).

## Supplementary Materials

The following are available online at http://www.mdpi.com/1996-1944/11/12/2450/s1, Figure S1: Loss (viscous) moduli *G*′(*ω*) of (a) neat polymers and (b–d) (70/30), (50/50), and (30/70) PLA/PVDF blends processed for different times as a function of frequency from 0.01 to 100 rad/s at a strain amplitude of 0.5% and a temperature of 200 °C under a nitrogen atmosphere, Figure S2: Complex viscosities $\left|\eta^{*}\left(\omega \right)\right|$ of the (70/30), (50/50), and (30/70) PLA/PVDF blends processed for (a) 5, (b) 7.5, and (c) 10 min as a function of frequency from 0.01 to 100 rad/s at a strain amplitude of 0.5% and a temperature of 200 °C under a nitrogen atmosphere, Figure S3: Complex viscosity of the neat polymers at a strain amplitude of 0.5% and a temperature of 200 °C under a nitrogen atmosphere. A Carreau–Yasuda fitting was utilized to obtain the zero-shear viscosity values required for calculation of the viscosity ratio.
