# Supplementary material for: Rheology–Microstructure Relationships in Melt-Processed Polylactide/Poly(vinylidene Fluoride) Blends
Source: Materials (Basel). 2018 Dec 3;11(12):2450. doi: 10.3390/ma11122450 (PMC6317166; doi:10.3390/ma11122450)
Supplement: Supplementary file 1 [file materials-11-02450-s001.pdf]

materials

Supplementary: Rheology–Microstructure Relationships in Melt-Processed Polylactide/Poly(Vinylidene Fluoride) Blends

Reza Salehiyan, Suprakas Sinha Ray, Florian J. Stadler and Vincent Ojijo

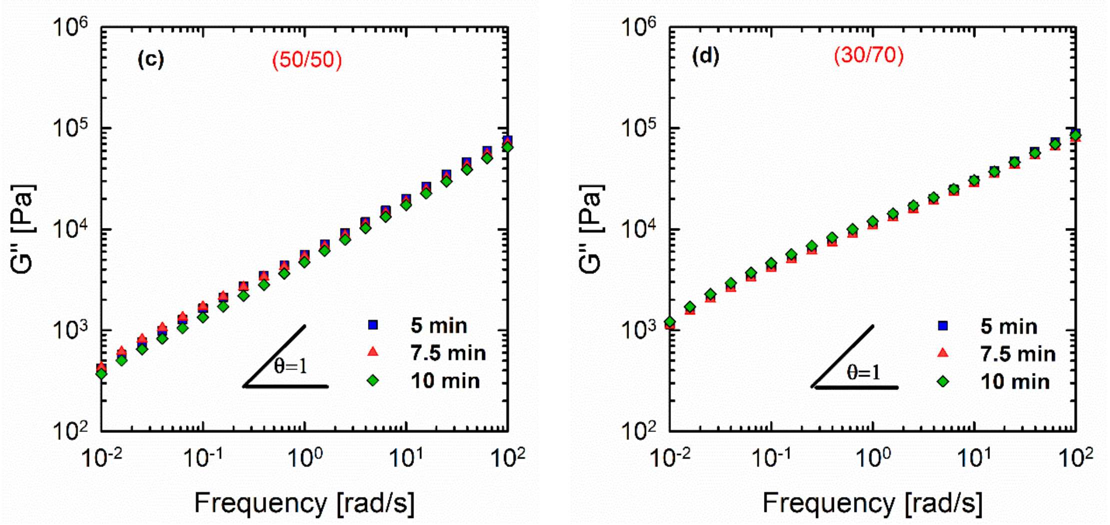

(a) (b)

Figure S1. Loss (viscous) moduli G′ (ω) of a (a) neat polymers and (b-d) (70/30), (50/50) and (30/70) PLA/PVDF blends processed for different times as a function of frequency from 0.01 to 100 rad/s at strain amplitude of 0.5% and temperature of 200 °C under nitrogen atmosphere.

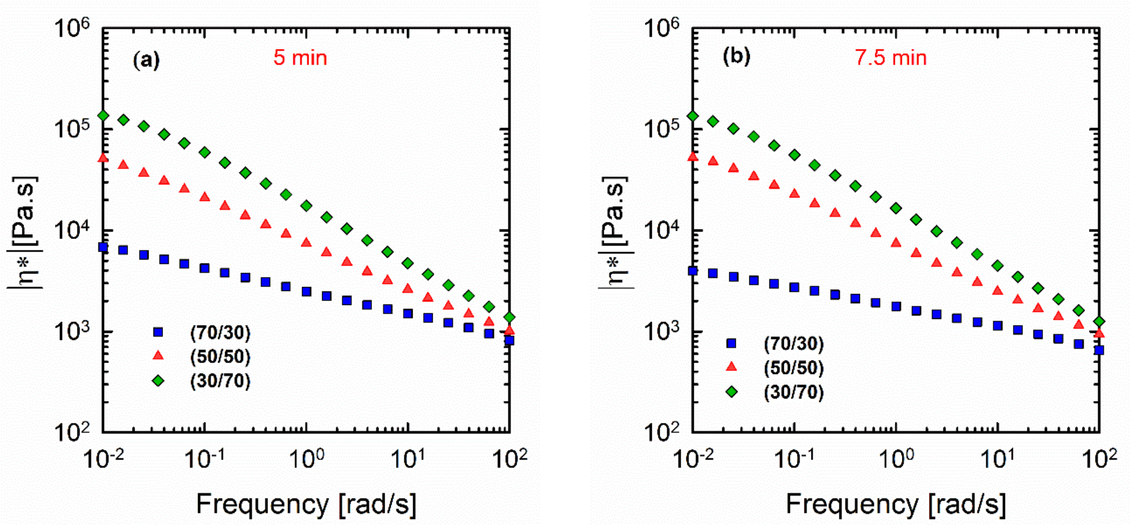

(a) (b)

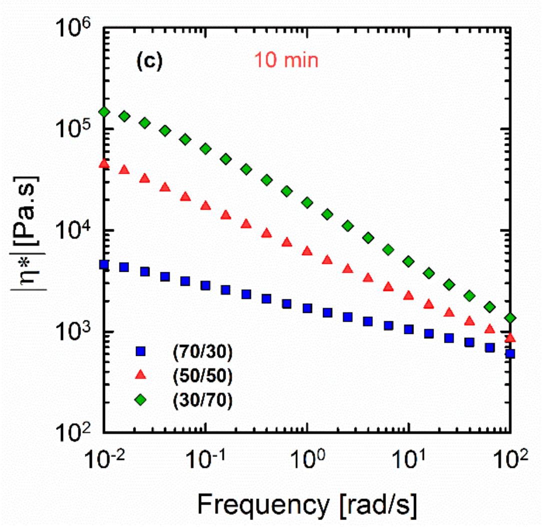

(c)

Figure S2. Complex viscosities $|\eta^*\ (\omega)|$ of the (70/30), (50/50) and (30/70) PLA/PVDF blends processed for (a) 5, (b) 7.5 and (c) 10 min as a function of frequency from 0.01 to 100 rad/s at strain amplitude of 0.5% and temperature of 200 °C under nitrogen atmosphere.

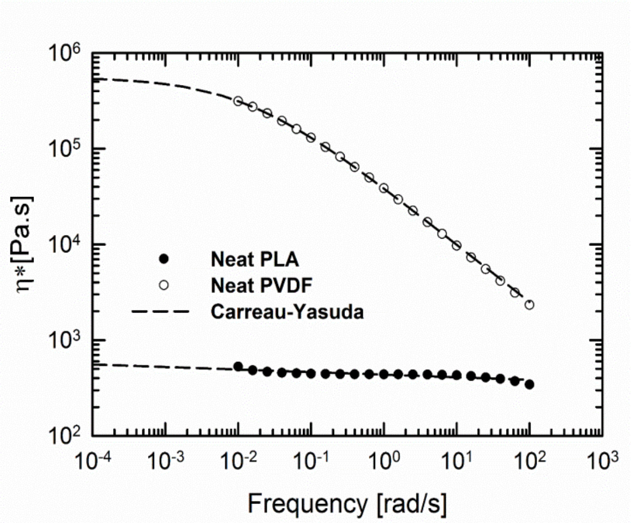

Figure S3. Complex viscosity of the neat polymers at strain amplitude of 0.5% and temperature of 200 °C under nitrogen atmosphere. Carreau-Yasuda fitting was utilized to obtain the zero-shear viscosity values required for calculation of viscosity ratio.

© 2018 by the authors. Submitted for possible open access publication under the terms and conditions of the Creative Commons Attribution (CC BY) license (http://creativecommons.org/licenses/by/4.0/).
